# Sarcoid-Like Granulomatous Disease: Pathologic Case Series in World Trade Center Dust Exposed Rescue and Recovery Workers

**DOI:** 10.3390/ijerph16050815

**Published:** 2019-03-06

**Authors:** Vasanthi R. Sunil, Jared Radbel, Sabiha Hussain, Kinal N. Vayas, Jessica Cervelli, Malik Deen, Howard Kipen, Iris Udasin, Robert Laumbach, Jag Sunderram, Jeffrey D. Laskin, Debra L. Laskin

**Affiliations:** 1 Ernest Mario School of Pharmacy, Rutgers University, Piscataway, NJ 08854, USA; kinalv5@pharmacy.rutgers.edu (K.N.V.); j.cervelli@pharmacy.rutgers.edu (J.C.); laskin@eohsi.rutgers.edu (D.L.L.); 2 Robert Wood Johnson Medical School, Rutgers University, New Brunswick, NJ 08901, USA; jr1106@rwjms.rutgers.edu (J.R.); sabiha.hussain@rutgers.edu (S.H.); malik.deen@rutgers.edu (M.D.); sunderja@rwjms.rutgers.edu (J.S.); 3 School of Public Health, Rutgers University, Piscataway, NJ 08854, USA; kipen@eohsi.rutgers.edu (H.K.); iu22@eohsi.rutgers.edu (I.U.); laumbach@eohsi.rutgers.edu (R.L.); jlaskin@eohsi.rutgers.edu (J.D.L.)

**Keywords:** WTC, fibrotic sarcoid, injury, inflammation, fibrosis

## Abstract

Sarcoid-like granulomatous diseases (SGD) have been previously identified in cohorts of World Trade Center (WTC) dust-exposed individuals. In the present studies, we analyzed lung and/or lymph node biopsies from patients referred to our clinic with suspected WTC dust-induced lung disease to evaluate potential pathophysiologic mechanisms. Histologic sections of lung and/or lymph node samples were analyzed for markers of injury, oxidative stress, inflammation, fibrosis, and epigenetic modifications. Out of seven patients examined, we diagnosed four with SGD and two with pulmonary fibrosis; one was diagnosed later with SGD at another medical facility. Patients with SGD were predominantly white, obese men, who were less than 50 years old and never smoked. Cytochrome b5, cytokeratin 17, heme oxygenase-1, lipocalin-2, inducible nitric oxide synthase, cyclooxygenase 2, tumor necrosis factor α, ADP-ribosylation factor-like GTPase 11, mannose receptor-1, galectin-3, transforming growth factor β, histone-3 and methylated histone-3 were identified in lung and lymph nodes at varying levels in all samples examined. Three of the biopsy samples with granulomas displayed peri-granulomatous fibrosis. These findings are important and suggest the potential of WTC dust-induced fibrotic sarcoid. It is likely that patient demographics and/or genetic factors influence the response to WTC dust injury and that these contribute to different pathological outcomes.

## 1. Introduction

Following the collapse of the World Trade Center (WTC) towers on 11 September, 2001, toxic dust was suspended in the air of lower Manhattan and Brooklyn. Over time, rescue and recovery workers exposed to WTC dust developed various respiratory diseases involving both the airways (asthma-like airway hyper-reactivity, bronchiolitis), and less commonly, the lung parenchyma (eosinophilic pneumonia, fibrosis, sarcoid-like granulomatous disease) [1,2]. Most notable is sarcoid-like granulomatous disease (SGD), which has been diagnosed at relatively high rates in cohorts of firefighters and rescue workers who have been followed since 2001 [3]. This condition is distinct from frank sarcoidosis which is a diagnosis of exclusion, once other known causes of granulomatous disease have been ruled out. It may be that WTC dust-induced SGD is a unique pathology, with potential for specific therapeutic interventions. 

Although WTC dust has been extensively characterized [4], little is known about the pathophysiologic mechanisms underlying the development of SGD and other lung pathologies. Studies in rodents have reported increased expression of genes associated with inflammation and oxidative stress in the lung following WTC dust exposure [5,6]. However, in these studies, granulomatous inflammation was not observed, making determination of the pathogenesis of WTC dust-induced SGD in humans, problematic.

In this report, we describe the pathologic findings in seven patients exposed to WTC dust who presented with ambiguous clinical pulmonary diagnoses. In all of the patients, we identified markers of injury, oxidative stress, inflammation, and epigenetic changes in lung and/or lymph nodes. Based on pathologic findings, five of the patients demonstrated evidence of SGD and three of these had peri-granulomatous fibrosis. These findings are important as they suggest the possibility of development of fibrosis in patients with WTC dust-induced SGD. This may lead to changes in clinical outcomes and treatment strategies.

## 2. Methods

### 2.1. Patients

Investigators at the Environmental and Occupational Health Sciences Institute (EOHSI) of Rutgers University are part of a New York/New Jersey consortium that has been following a cohort of rescue and recovery workers exposed to dust and other materials at the WTC site. These individuals included rescue, recovery, debris-cleanup, law enforcement, and related support service workers, and volunteers in lower Manhattan (south of Canal St.), the Staten Island Landfill, and/or the barge loading piers, who worked on-site for at least 4 h/day between 11 and 14 September, 2001, for at least 24 h during September 2001, or for at least 80 h total time between September and December 2001 [7]. From among approximately 2000 patients followed at EOHSI, 7 were referred for biopsy between 2007 and 2011, if a final pulmonary diagnosis despite symptom review and full evaluation including pulmonary function testing (PFT) and computerized tomography (CT) scan interpretation required tissue analysis. Patient demographics were collected, including WTC dust exposure levels as described by Wisnivesky et al. [2]. Lung and/or mediastinal lymph node specimens were obtained for both histopathologic evaluation and immunohistochemical staining. All subjects gave informed consent for inclusion before they participated in the study. The study was conducted in accord with the Declaration of Helsinki, and the protocol was approved by the Ethics Committee of the Rutgers Institutional Review Board.

### 2.2. Pulmonary Function Testing (PFT)

PFTs were performed using NSpire Health pulmonary functioning testing devices (NSpire Health, Longmont, CO, USA), that were calibrated in the Rutgers University Robert Wood Johnson Medical School Pulmonary Function Laboratory. Test results were interpreted according to American Thoracic Society guidelines and results collected for each patient [8]. 

### 2.3. CT Scans

Tidal breathing CT was performed from lung apex to lung base without the use of intravenous contrast material. All results were interpreted by board-certified radiologists.

### 2.4. Sample Collection, Histology and Immunohistochemistry

We collected lung samples by transbronchial biopsy (TBB) from 3 patients, and by video-assisted thoracoscopy (VATS) from 2 patients. Three patients had mediastinal lymph node biopsies obtained via mediastinoscopy (Med). Samples were fixed in 3% paraformaldehyde for 4 h and then transferred to 50% ethanol. Histological sections (4 μm) were prepared and stained with hematoxylin and eosin (H and E) or Masson’s trichrome stain. Tissues were analyzed by a board-certified pulmonary pathologist (M. Deen) for the extent of inflammation, including macrophage and neutrophil localization, alterations in alveolar epithelial barriers, fibrin deposition, edema, granuloma formation, and fibrosis. Representative images were acquired at high resolution (magnification 60×) using an Olympus VS120 Virtual Microscopy System, scanned and viewed using OlyVIA version 2.6 software (Olympus Life Sciences, Center Valley, PA, USA). 

For immunohistochemistry, tissue sections were deparaffinized with xylene (4 min, ×2) followed by decreasing concentrations of ethanol (100%–50%) and finally, water. After antigen retrieval using citrate buffer (10.2 mM sodium citrate, 0.05% Tween 20, pH 6.0) and quenching of endogenous peroxidase with 3% H_2_O_2_ for 10–30 min, sections were incubated for 1–4 h at room temperature with 5–100% goat serum to block nonspecific binding. This was followed by overnight incubation at 4 °C with rabbit IgG or rabbit polyclonal anti-cytochrome b5 (1:250, Abcam, Cambridge, MA, USA), anti-cytokeratin 17 (1:1000, Abcam), anti-heme oxygenase (HO)-1 (1:650, Enzo Life Sciences, Farmingdale, NY, USA), anti-lipocalin (Lcn)-2 (1:250, Abcam), anti-inducible nitric oxide synthase (iNOS) (1:500, Abcam), anti-cyclooxygenase (COX)-2 (1:100, Abcam), anti-tumor necrosis factor (TNF)α (1:50, Abcam), anti-ADP-ribosylation factor-like GTPase (ARL)11 (1:100, Bioss Antibodies, Woburn, MA, USA), anti-mannose receptor (MR)-1 (1:500, Abcam), anti-galectin (Gal)-3 (1:400, R&D Systems, Minneapolis, MN, USA), anti-transforming growth factor (TGF)β (1:50, Abcam), anti-histone H3 (1:50, Cell Signaling, Danvers, MA, USA), or anti-mono-methyl histone H3K4 (1:100, Cell Signaling) antibodies. Sections were then incubated with biotinylated secondary antibody (Vector Labs, Burlingame, CA, USA) for 30 min at room temperature. Binding was visualized using a DAB (3,3’ diaminobenzidine) peroxidase substrate kit (Vector Labs). Sections of lung and/or lymph nodes from all 7 subjects were analyzed for each antibody. 

## 3. Results

### 3.1. Patient Demographics and Diagnoses 

Patient demographics are presented in Table 1. The patients were predominantly male (100%), white (non-Hispanic; 100%), and former smokers (57%). Based on pathological findings (see below), patients were divided into two groups; those with SGD (*n* = 5) and those with other pathology (*n* = 2). Of the patients with SGD, 100% were white males, overweight or obese (BMI = 25–29 (20%); BMI ≥ 30 (80%)), 80% were <50 years old (mean age 43.5), and 60% never smoked (Table 1). Four of the five patients with SGD had an intermediate WTC dust exposure level, and one had a high level of exposure [2]. The two non-SGD patients had high WTC dust exposure levels (Table 1). The clinical, radiographic and pathologic characteristics of patients undergoing biopsy are presented in Table 2. The reasons for biopsy in patients with SGD (patients #1–5) were mediastinal/hilar adenopathy and pulmonary nodules (Table 2). Other patients displayed a mix of lymphadenopathy, fibrosis, nodules, and emphysema. Of patients with SGD, patients #1 and #2 had normal pulmonary function, while patients #3 and #4 displayed restrictive physiology and patient #5 had mixed obstruction and restriction (Table 3). The two patients without SGD (#6 and #7) had restrictive physiology (Table 3).

### 3.2. Pathological Results

**Figure 1:**
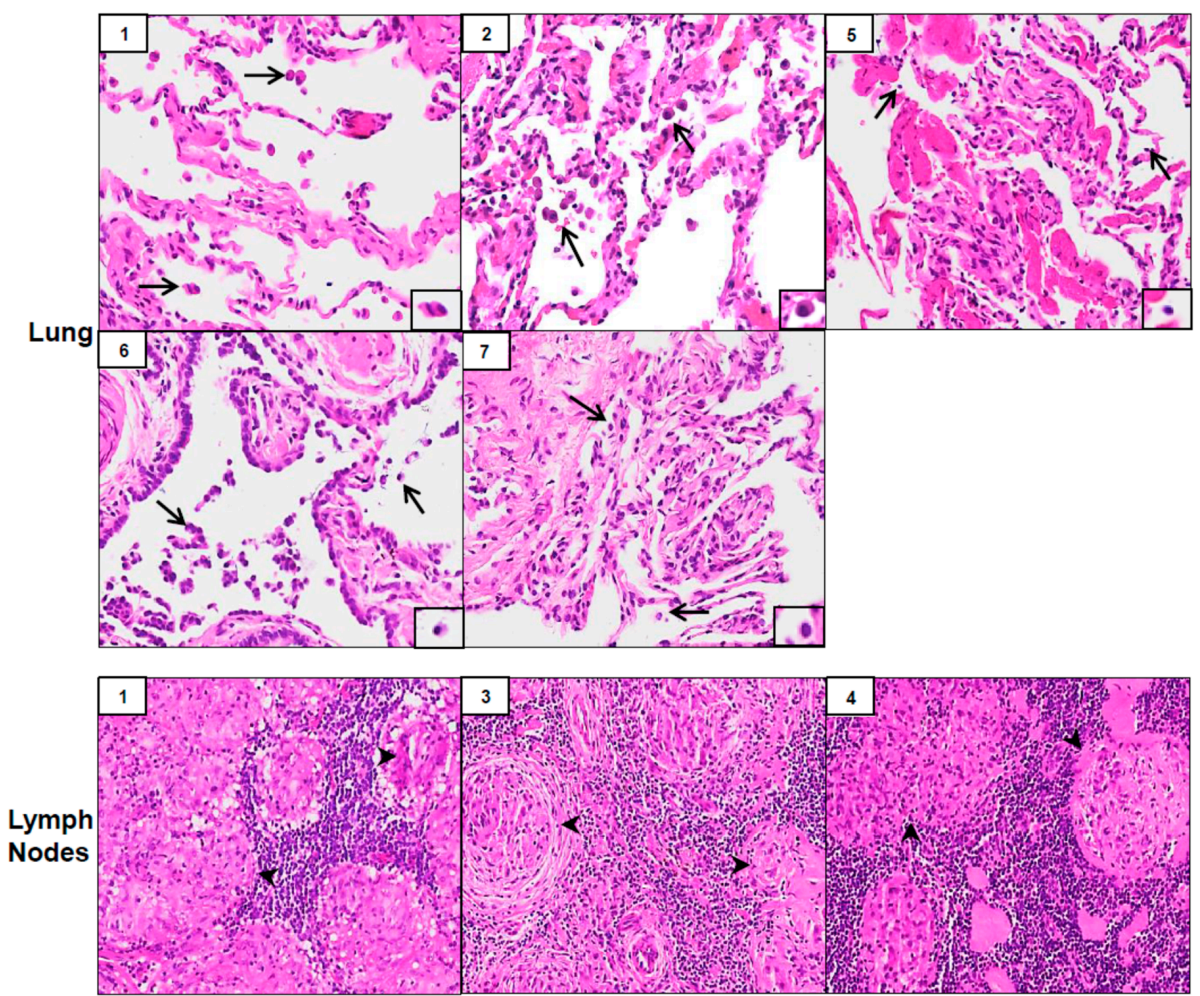
Histology of lung and lymph nodes. Biopsies of lung (top and middle panels) and lymph nodes (bottom panels), collected from World Trade Center (WTC) rescue and recovery workers, were sectioned and stained with Hematoxylin and Eosin (H&E). Inset, patient number. Magnification, 20×; arrows, alveolar macrophages; arrowheads, lymphocytes.

**Figure 2:**
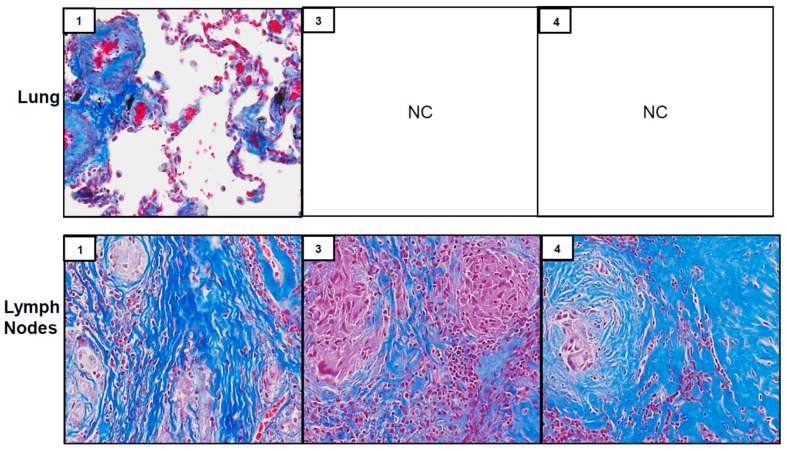
Trichrome staining of lung and lymph nodes. Biopsies of lung (top panel) and lymph nodes (bottom panels), collected from WTC rescue and recovery workers were sectioned and stained with Masson’s trichrome. Inset, patient number; NC, tissue not collected. Magnification, 13.2×.

Biopsy samples from three of the seven patients (patients #1, #3 and #4) showed well-formed non-caseating granulomas in lymph nodes suggesting SGD; patient #2 exhibited scant, poorly formed granuloma in the lung (Table 2 and Figure 1). In two of the patients (#3 and #4), only lymph nodes were biopsied; in one patient (#2), only lung parenchyma was biopsied. In patient #1, both lymph nodes and lung were biopsied, and both tissue types exhibited non-caseating granulomas (Figure 1). Masson’s trichrome staining of tissues revealed the presence of well-formed granulomas and evidence of peri-granulomatous fibrosis in lymph nodes of patients #1, #3 and #4 (Figure 2). Of the other three patients without granulomatous changes (#5–#7), pathological diagnoses in the lung included mild fibrosis, bronchial inflammation and acute and chronic lung injury (Table 2 and Figure 1). Patient #5 was diagnosed later with SGD at another medical facility after obtaining additional tissue via VATS.

### 3.3. Expression of Markers of Injury, Oxidative Stress, Inflammation, Fibrosis, and Epigenetic Changes

**Figure 3:**
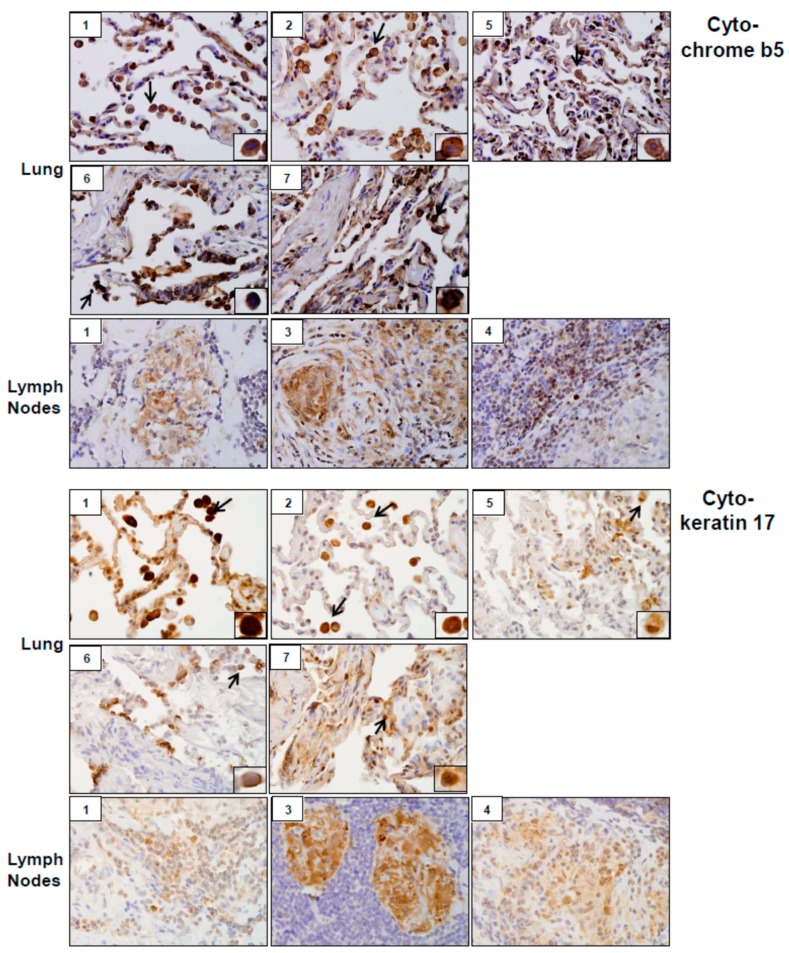
Cytochrome b5 and cytokeratin 17 expression in lung and/or lymph nodes collected from WTC rescue and recovery workers. Sections were immunostained with antibody to cytochrome b5 or cytokeratin 17. Binding was visualized using a 3,3’ diaminobenzidine (DAB) peroxidase substrate kit. One representative section from each patient is shown. Inset, patient number. Magnification, 60×; arrows, alveolar macrophages.

**Figure 4:**
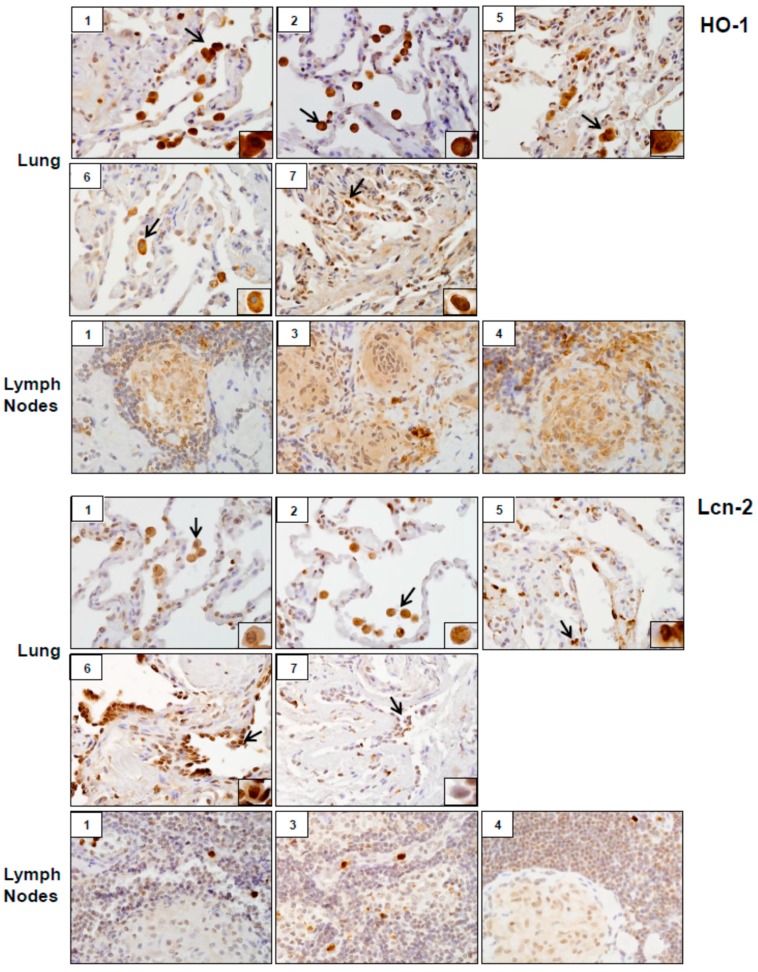
Hemeoxygenase (HO)-1 and Lipocalin (Lcn)-2 expression in lung and/or lymph nodes collected from WTC rescue and recovery workers. Sections were immunostained with antibody to HO-1 or Lcn-2. Binding was visualized using a DAB peroxidase substrate kit. One representative section from each patient is shown. Inset, patient number. Magnification, 60×; arrows, alveolar macrophages.

**Figure 5:**
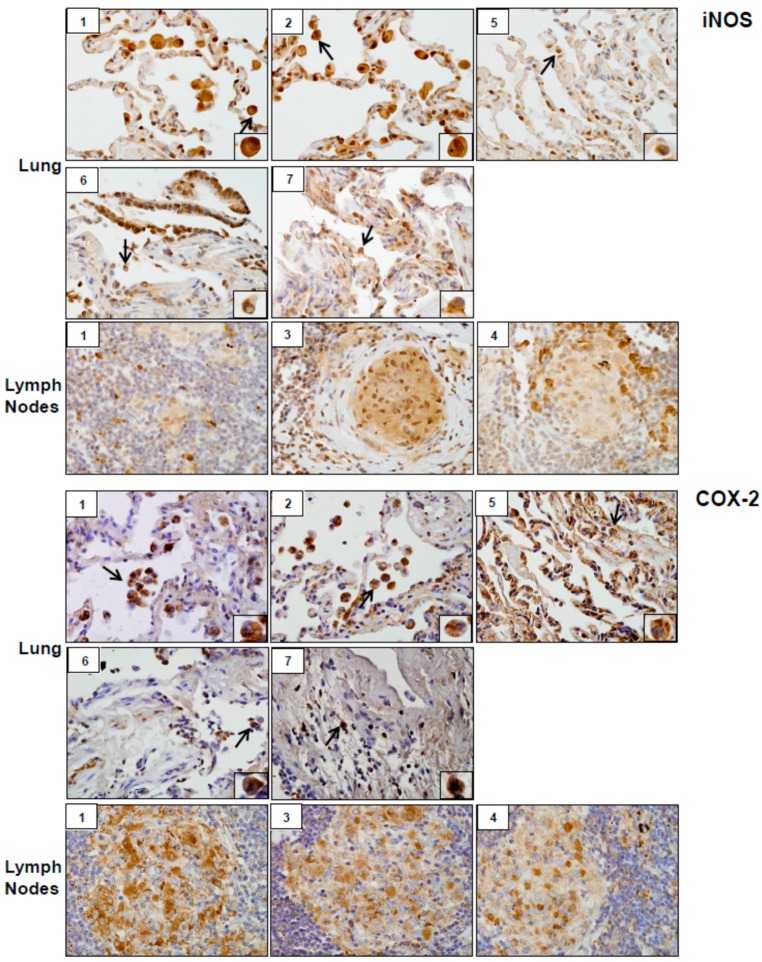
Inducible nitric oxide synthase (iNOS) and Cyclooxygenase (COX)-2 expression in lung and/or lymph nodes collected from WTC rescue and recovery workers. Sections were immunostained with antibody to iNOS or COX-2. Binding was visualized using a DAB peroxidase substrate kit. One representative section from each patient is shown. Inset, patient number. Magnification, 60×; arrows, alveolar macrophages.

**Figure 6:**
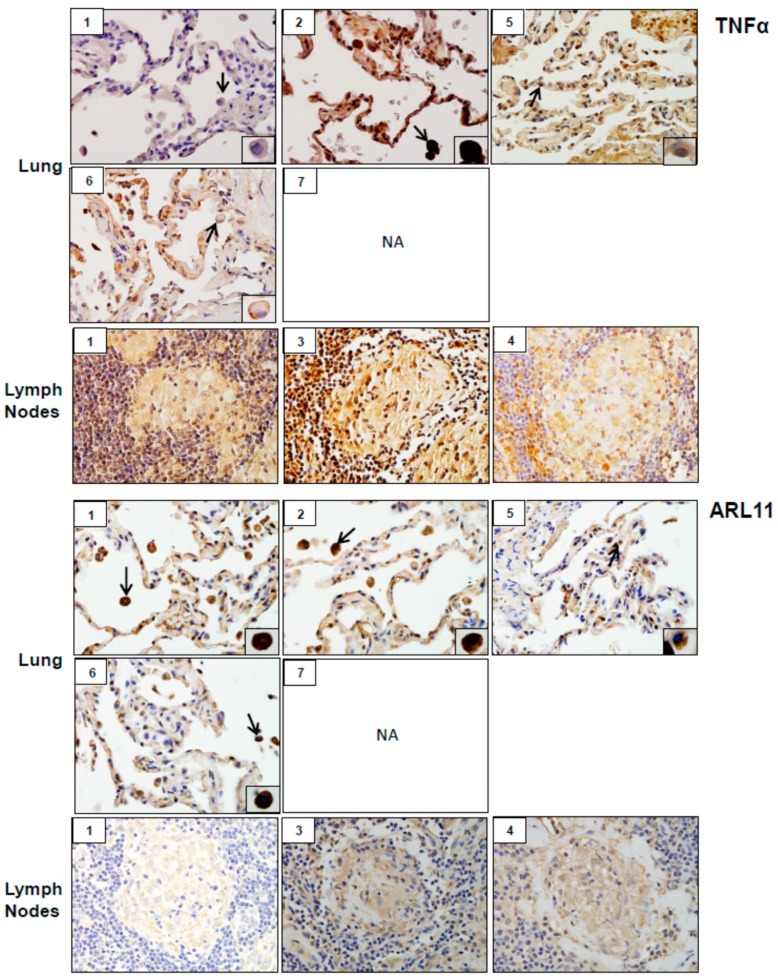
Tumor necrosis factor (TNF)α and ADP-ribosylation factor-like GTPase (ARL)11 expression in lung and/or lymph nodes collected from WTC rescue and recovery workers. Sections were immunostained with antibody to TNFα or ARL11. Binding was visualized using a DAB peroxidase substrate kit. One representative section from each patient is shown. Inset, patient number; NA, tissue not available. Magnification, 60×; arrows, alveolar macrophages.

**Figure 7:**
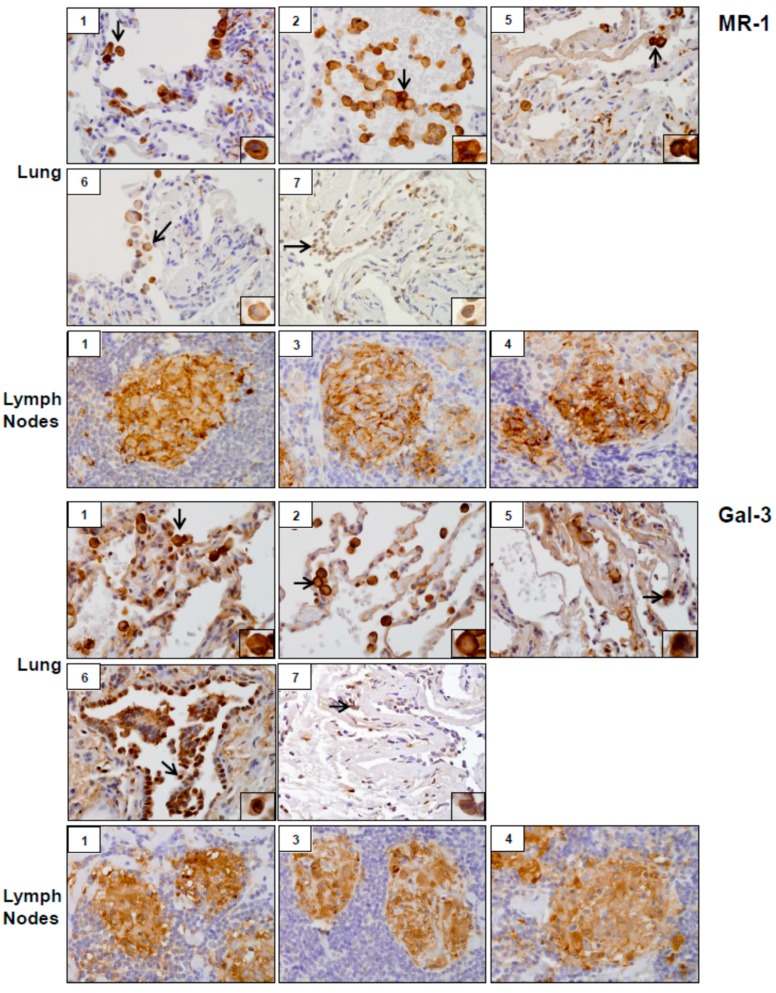
Mannose receptor (MR)-1 and Galectin (Gal)-3 expression in lung and/or lymph nodes collected from WTC rescue and recovery workers. Sections were immunostained with antibody to MR-1 or Gal-3. Binding was visualized using a DAB peroxidase substrate kit. One representative section from each patient is shown. Inset, patient number. Magnification, 60×; arrows, alveolar macrophages.

**Figure 8:**
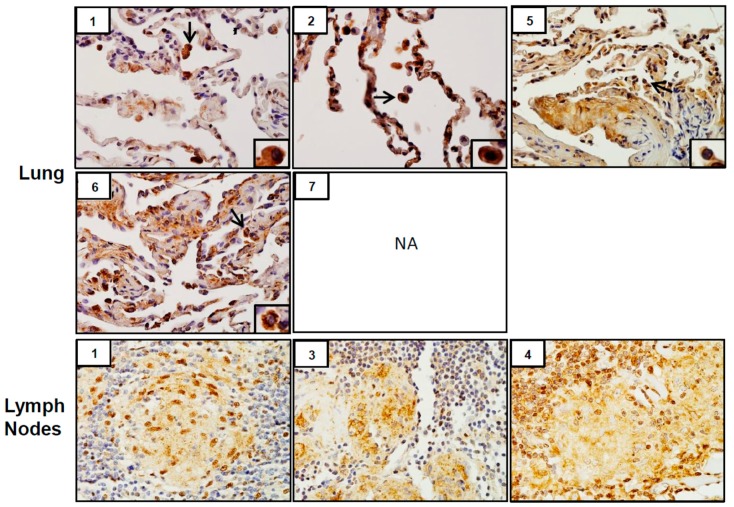
Transforming growth factor (TGF)β expression in lung and/or lymph nodes collected from WTC rescue and recovery workers. Sections were immunostained with antibody to TGFβ. Binding was visualized using a DAB peroxidase substrate kit. One representative section from each patient is shown. Inset, patient number; NA, tissue not available. Magnification, 60×; arrows, alveolar macrophages.

**Figure 9:**
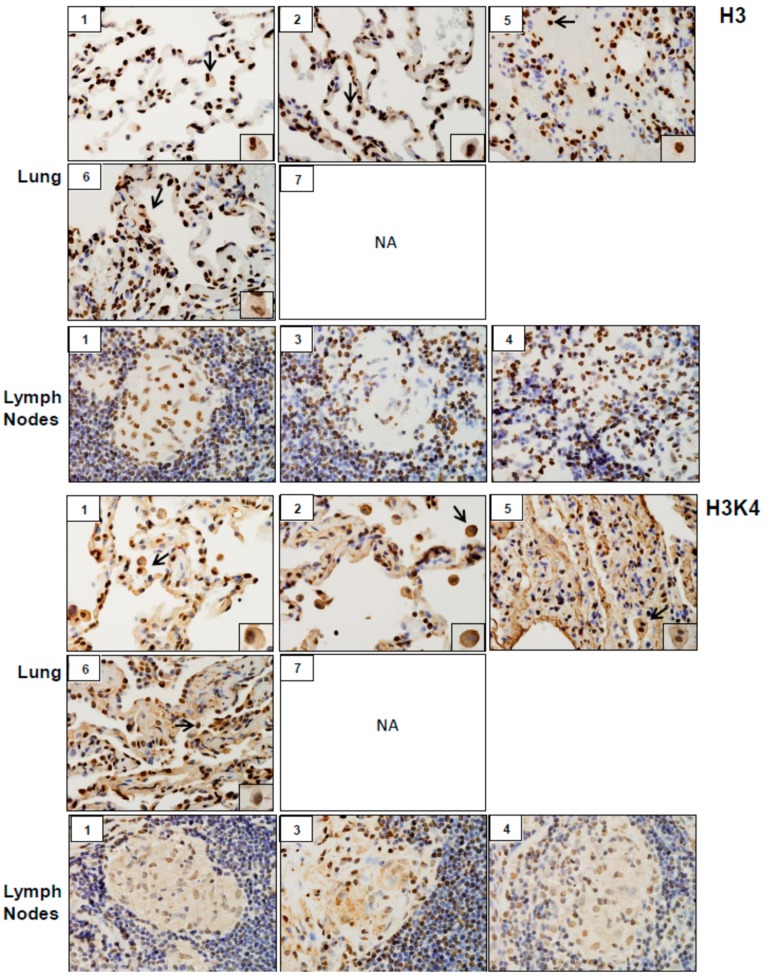
Histone H3 and methylated (K4) histone H3K4 expression in lung and/or lymph nodes collected from WTC rescue and recovery workers. Sections were immunostained with antibody to H3 or H3K4. Binding was visualized using a DAB peroxidase substrate kit. One representative section from each patient is shown. Inset, patient number; NA, tissue not available. Magnification, 60×; arrows, alveolar macrophages.

Analysis of tissue sections by immunohistochemistry revealed the presence of cytochrome b5 and cytokeratin 17, markers of injury in lung (patients #1, # 2, #5, #6 and #7) and lymph nodes (patients #1, #3 and #4) (Figure 3). Whereas cytochrome b5 expression was uniformly distributed throughout the lung, including alveolar macrophages and epithelial cells, cytokeratin 17 was more prominent in alveolar macrophages. However, the intensity of expression of both of these markers varied between patients. Markers of oxidative stress including HO-1 and Lcn-2 were upregulated in alveolar macrophages and lymph node biopsies from all patients (Figure 4). As observed with cytochrome b5 and cytokeratin 17, the intensity of expression varied between patients. We also noted upregulation of the proinflammatory proteins iNOS, COX-2, TNFα, and ARL11 to varying degrees in alveolar macrophages and lymph nodes in all patient samples examined (Figure 5 and Figure 6). Anti-inflammatory/pro-fibrotic markers of macrophage activation, including MR-1, the galactoside-binding lectin, Gal-3, and TGFβ were also upregulated (Figure 7 and Figure 8). Additionally, histone H3 and methylated (K4) histone H3K4, epigenetic markers of inflammation/fibrosis were identified in lung and lymph nodes with varying intensities in all patient samples examined (Figure 9).

## 4. Discussion 

The present studies report that five of the seven patients referred to our clinic for diagnostic evaluation exhibited SGD, as evidenced by non-caseating granulomas in lung and/or lymph nodes. SGD has previously been described among a subset of WTC rescue and recovery workers [9,10]. Mechanisms underlying the development of this disease remain unknown [11,12,13,14,15]. Non-caseating granuloma formation is a type of foreign body reaction which involves trapping of remnants of foreign materials that cannot be degraded and/or destroyed by macrophages [16]. The appearance of non-caseating granulomas in WTC dust-exposed patients is in line with findings that WTC dust contained organic and inorganic particles which have been implicated in the development of granulomatous pulmonary disease [17].

Inflammation and oxidative stress are common responses to inhalation of particles and fibers including silica and asbestos, components of WTC dust, and they are thought to be involved in pulmonary disease pathogenesis [18,19,20]. As a first step in elucidating WTC dust-induced SGD mechanisms, we analyzed the expression of markers of oxidative stress, inflammation, and injury in lung and/or lymph nodes of WTC dust-exposed patients followed in our clinic. Lung and/or lymph node samples from all patients examined stained positively for markers of pro-inflammatory M1 macrophages including iNOS, COX-2, TNFα and ARL11 [21,22,23]. iNOS and COX-2 mediate the production of reactive nitrogen species and proinflammatory eicosanoids, respectively. These mediators are known to contribute to M1 macrophage activation and lung injury induced by diverse toxicants, and they may play a similar role in the pathogenic response to WTC dust [21]. In this regard, COX-2 has been reported to be upregulated by silica, a component of WTC dust, in rodents, cultured fibroblasts, and in sarcoid granulomas [4,18,24,25,26]. ARL11 has recently been identified as a regulator of proinflammatory macrophage activation and TNFα release [22,27]. TNFα has been implicated in lung injury induced by silica [26], suggesting a potential mechanism of disease pathogenesis following WTC dust exposure. 

Previous studies showed that WTC dust induces oxidative stress [5,6]. Consistent with these reports, tissue samples from all patients examined were found to stain positively for the oxidative stress markers, HO-1 and Lcn-2. In addition to their anti-oxidant activity, these proteins promote anti-inflammatory responses. Macrophage Lcn-2 has previously been shown to reduce granulomatous inflammation in mycobacterial pulmonary infections [28]. Upregulation of HO-1 and Lcn-2 in granulomas of WTC dust-exposed patients may reflect a compensatory attempt to limit inflammation and granuloma progression. Increases in proinflammatory macrophage mediators and oxidative stress were associated with lung damage as evidenced by upregulation of cytochrome b5 and cytokeratin 17 in macrophages and epithelial cells. Similar increases in these proteins have been reported in acute lung injury induced by inhaled ozone and *S. aureus* enterotoxin [23,29,30]. 

The activity of proinflammatory/cytotoxic M1 macrophages is balanced by anti-inflammatory/pro-fibrotic M2 macrophages, which downregulate inflammation and initiate wound repair. However, when overactivated, M2 macrophages promote fibrosis [21]. Findings that macrophages in histologic sections of WTC dust-exposed patients stained positively for MR-1 and Gal-3 suggest M2 macrophage activation [31]. This is in accord with findings that Gal-3 promotes M2 polarization of macrophages and contributes to lung fibrosis [32,33]. 

Coordinate with the presence of M2 macrophages, we found that tissue samples stained positively for the pro-fibrotic protein TGF β. Of note, patients with well-formed granulomas (#1, #3 and #4) also exhibited peri-granulomatous fibrosis, as evidenced by trichrome staining. Fibrotic sarcoidosis is characterized pathologically as fibrotic destruction at sites of prior granulomatous inflammation [34]. The granulomas are thought to function as a nidus for the development of fibrosis that can encompass larger areas of the respiratory tract, resulting in collagen deposition in broncho-vascular tracts and interlobular septae, cystic distortion and honeycombing of the lung [35,36]. It remains to be determined if peri-granulomatous fibrosis in our series is an early indicator of progressive fibrotic disease in patients with WTC dust-induced SGD. The most recent follow up of a large WTC dust-exposed firefighter cohort with SGD did not report clinical evidence of fibrotic disease [13]. Our pathological series suggests that fibrosis may develop in these patients with time. This is important as the development of fibrosis in sarcoidosis represents a significant change in the clinical course that is associated with increased morbidity and mortality [34,35]. 

It is important to note that expression of inflammatory proteins was observed in all patients exposed to WTC dust, regardless of the presence of granulomas. As we did not have controls to assess for comparison, a causal link has not been established. It is likely that specific patient demographics, such as smoking, obesity, age, and/or genetic/epigenetic factors influence the immune response to WTC dust injury, contributing to different pathological outcomes. In this regard, our patients with SGD were predominantly non-smokers, obese and <50 years of age at diagnosis. This is similar to the demographic profile of larger cohorts of New York City firefighters and first responders exposed to WTC dust [3,13]. Evidence suggests that sarcoidosis is more likely to develop in young, obese, non-smokers [37,38]. These factors are known to affect the adaptive immune response by inducing T-cell differentiation towards a Th1 phenotype [39,40,41,42]. This may contribute to the development of WTC dust-induced SGD. Lung and lymph node biopsies in our cohort, also stained positively for histone H3 and H3K4, suggesting the possibility that epigenetic factors may also contribute to SGD. 

All five patients with SGD had intermediate/high exposure to WTC dust. Previous studies demonstrated a strong correlation between WTC dust exposure levels and the development of lung disease with very high exposure levels causing the most disease [2]. Our studies suggest that intermediate levels of WTC dust exposure may be a sufficient risk factor for developing SGD with fibrosis. It is also possible that heterogeneity in WTC dust, such as composition (silica, metal) and particle size, affect the pathologic response [36]. These possibilities require further investigation. 

### Limitations

In this case series studies, we are limited in any inferences we can make about associations, since our sample size was small and non-random, and we did not have case-matched controls.

## 5. Conclusions

In summary, our studies demonstrate the presence of SGD in five of seven patients exposed to intermediate/high levels of WTC dust. Lung and/or lymph nodes exhibited signs of oxidative stress, inflammation, tissue damage, and fibrosis. This was associated with upregulation of proinflammatory and pro-fibrotic markers and epigenetic alterations. Our observations of peri-granulomatous fibrosis in some of the patients with SGD are novel. Further studies are required to elucidate the precise mechanisms underlying this pathology in subsets of WTC dust-exposed patients.

## Figures and Tables

**Table 1:** Demographic characteristics of patients undergoing biopsy.

Characteristics	Total Number (%)	SGD Number (%)	Other Diseases Number (%)
*Gender*			
Male	7 (100)	5 (100)	2 (100)
Female	0 (0)	0 (0)	0 (100)
*Age*			
<50	4 (57)	4 (80)	(0)
50–59	1 (14)	1 (20)	0 (0)
60–69	2 (29)	0 (0)	2 (100)
*Race/Ethnicity*			
White (non-Hispanic)	7 (100)	5 (100)	2 (100)
Black (non-Hispanic)	0 (0)	0 (0)	0 (0)
Hispanic	0 (0)	0 (0)	0 (0)
*Smoking Status*			
Current	0 (0)	0 (0)	0 (0)
Past	4 (57)	2 (40)	2 (100)
Never	3 (43)	3 (60)	0 (0)
*BMI (kg/m^2^)*			
< 25	0 (0)	0 (0)	0 (0)
25–29	3 (43)	1 (20)	2 (100)
30–39	4 (57)	4 (80)	0 (0)
*WTC Exposure Category* [2]			
Very High	0 (0)	0 (0)	0 (0)
High	3 (43)	1 (20)	2 (100)
Intermediate	4 (57)	4 (80)	0 (0)
Low	0 (0)	0 (0)	0 (0)

Abbreviations: SGD, sarcoid-like granulomatous disease; BMI, body mass index; WTC, World Trade Center.

**Table 2:** Clinical, radiographic and pathologic presentation of patients.

PT#	Reason for Referral	CT Impression	Biopsy	Lung Pathology	Lymph Node Pathology	Diagnosis
1	Lung nodules, mediastinal LAD	Mediastinal LAD, ground glass, sub-centimeter peripheral nodules, very mild fibrotic changes	VATS and Med	Bilateral upper lobe: Non-caseating granulomas with surrounding fibrosis	Non-caseating granulomas with surrounding fibrosis	SGD
2	Chronic cough and dyspnea	Multiple nodules	VATS	Right upper and lower lobe: Scant, poorly formed granulomas	NC	SGD
3	Hilar and mediastinal LAD and chronic bronchitis	Hilar and mediastinal LAD, apical pleural thickening	Med	NC	Non-caseating granulomas with surrounding fibrosis	SGD
4	Hilar LAD	Mediastinal and hilar LAD with bilateral pleural-based nodules	Med	NC	Non-caseating granuloma with surrounding fibrosis	SGD
5	Dyspnea and fatigue	Pulmonary nodules with mediastinal LAD	TBB	Right lower lobe: Focal areas of lung injury. (proteinaceous exudate with fibrin deposit)	NC	SGD (Diagnosed later at another medical facility)
6	Cough, dyspnea	Punctate calcified granulomas in lower lobes, bronchiectasis, mild intralobular septal thickening, scattered ground glass, mediastinal LAD	TBB	Left lower lobe: Focal interstitial fibrosis	NC	Pulmonary fibrosis and adenocarcinoma of the lung
7	Cough, dyspnea	Centrilobular emphysema, interstitial fibrosis. No adenopathy	TBB	Right upper lobe Unremarkable.	NC	Pulmonary fibrosis

Abbreviations: PT#, patient number; CT, computerized tomography; LAD, lymphadenopathy; VATS, video-assisted thoracoscopy; Med, mediastinoscopy; TBB, trans-bronchial biopsy; SGD, sarcoid-like granulomatous disease; NC, sample not collected.

**Table 3:** Pulmonary function test findings in patients undergoing biopsy.

PT#	Spirometry			Lung Volumes		DLCO (%)	Impression
	FEV_1_ (%)	FVC (%)	FEV_1_/FVC (%)	VC (%)	TLC (%)		
1	98	100	97	98	93	103	WNL
2	100	104	96	103	109	104	WNL
3	88	84	104	81	82	85	Mild RTLD
4	98	94	104	83	73	69	Mild RILD
5	42	58	72	51	62	69	Severe Mixed OLD & RILD
6	98	87	112	80	78	57	Mild RILD
7	80	77	104	80	77	86	Mild RTLD

Abbreviations: PT#: patient number; FEV_1_: forced expiratory volume in 1 s; FVC: forced vital capacity; VC: vital capacity; TLC: total lung capacity; DLCO: diffusing capacity for carbon monoxide; WNL: within normal limits; RTLD: restrictive thoracic lung disease; RILD: restrictive interstitial lung disease; OLD: obstructive lung disease. Bolded values were below normal confidence intervals.

## References

[1] Guidotti T.L., Prezant D., de la Hoz R.E., Miller A. (2011). The evolving spectrum of pulmonary disease in responders to the World Trade Center tragedy. Am. J. Ind. Med..

[2] Wisnivesky J.P., Teitelbaum S.L., Todd A.C., Boffetta P., Crane M., Crowley L., de la Hoz R.E., Dellenbaugh C., Harrison D., Herbert R. (2011). Persistence of multiple illnesses in World Trade Center rescue and recovery workers: A cohort study. Lancet.

[3] Webber M.P., Yip J., Zeig-Owens R., Moir W., Ungprasert P., Crowson C.S., Hall C.B., Jaber N., Weiden M.D., Matteson E.L. (2017). Post-9/11 sarcoidosis in WTC-exposed firefighters and emergency medical service workers. Respir. Med..

[4] Lioy P.J., Weisel C.P., Millette J.R., Eisenreich S., Vallero D., Offenberg J., Buckley B., Turpin B., Zhong M., Cohen M.D. (2002). Characterization of the dust/smoke aerosol that settled east of the World Trade Center (WTC) in lower Manhattan after the collapse of the WTC 11 September 2001. Environ. Health Perspect..

[5] Cohen M.D., Vaughan J.M., Garrett B., Prophete C., Horton L., Sisco M., Kodavanti U.P., Ward W.O., Peltier R.E., Zelikoff J. (2015). Acute high-level exposure to WTC particles alters expression of genes associated with oxidative stress and immune function in the lung. J. Immunotoxicol..

[6] Sunil V.R., Vayas K.N., Fang M., Zarbl H., Massa C., Gow A.J., Cervelli J.A., Kipen H., Laumbach R.J., Lioy P.J. (2017). World Trade Center (WTC) dust exposure in mice is associated with inflammation, oxidative stress and epigenetic changes in the lung. Exp. Mol. Pathol..

[7] Herbert R., Moline J., Skloot G., Metzger K., Baron S., Luft B., Markowitz S., Udasin I., Harrison D., Stein D. (2006). The World Trade Center disaster and the health of workers: Five-year assessment of a unique medical screening program. Environ. Health Perspect..

[8] Miller M.R., Hankinson J., Brusasco V., Burgos F., Casaburi R., Coates A., Crapo R., Enright P., van der Grinten C.P., Gustafsson P. (2005). Standardisation of spirometry. Eur. Respir. J..

[9] Crowley L.E., Herbert R., Moline J.M., Wallenstein S., Shukla G., Schechter C., Skloot G.S., Udasin I., Luft B.J., Harrison D. (2011). “Sarcoid like” granulomatous pulmonary disease in World Trade Center disaster responders. Am. J. Ind. Med..

[10] Izbicki G., Chavko R., Banauch G.I., Weiden M.D., Berger K.I., Aldrich T.K., Hall C., Kelly K.J., Prezant D.J. (2007). World Trade Center “sarcoid-like” granulomatous pulmonary disease in New York City Fire Department rescue workers. Chest.

[11] Chen E.S., Song Z., Willett M.H., Heine S., Yung R.C., Liu M.C., Groshong S.D., Zhang Y., Tuder R.M., Moller D.R. (2010). Serum amyloid A regulates granulomatous inflammation in sarcoidosis through Toll-like receptor-2. Am. J. Respir. Crit. Care Med..

[12] Facco M., Cabrelle A., Calabrese F., Teramo A., Cinetto F., Carraro S., Martini V., Calzetti F., Tamassia N., Cassatella M.A. (2015). TL1A/DR3 axis involvement in the inflammatory cytokine network during pulmonary sarcoidosis. Clin. Mol. Allergy.

[13] Hena K.M., Yip J., Jaber N., Goldfarb D., Fullam K., Cleven K., Moir W., Zeig-Owens R., Webber M.P., Spevack D.M. (2018). Clinical Course of Sarcoidosis in World Trade Center-Exposed Firefighters. Chest.

[14] Schnerch J., Prasse A., Vlachakis D., Schuchardt K.L., Pechkovsky D.V., Goldmann T., Gaede K.I., Muller-Quernheim J., Zissel G. (2016). Functional Toll-Like Receptor 9 Expression and CXCR3 Ligand Release in Pulmonary Sarcoidosis. Am. J. Respir. Cell Mol. Biol..

[15] Wiken M., Idali F., Al Hayja M.A., Grunewald J., Eklund A., Wahlstrom J. (2010). No evidence of altered alveolar macrophage polarization, but reduced expression of TLR2, in bronchoalveolar lavage cells in sarcoidosis. Respir. Res..

[16] Valeyre D., Prasse A., Nunes H., Uzunhan Y., Brillet P.Y., Muller-Quernheim J. (2014). Sarcoidosis. Lancet.

[17] Safirstein B.H., Klukowicz A., Miller R., Teirstein A. (2003). Granulomatous pneumonitis following exposure to the World Trade Center collapse. Chest.

[18] Fubini B., Hubbard A. (2003). Reactive oxygen species (ROS) and reactive nitrogen species (RNS) generation by silica in inflammation and fibrosis. Free Radic. Biol. Med..

[19] Rimal B., Greenberg A.K., Rom W.N. (2005). Basic pathogenetic mechanisms in silicosis: Current understanding. Curr. Opin. Pulm. Med..

[20] Valavanidis A., Vlachogianni T., Fiotakis K., Loridas S. (2013). Pulmonary Oxidative Stress, Inflammation and Cancer: Respirable Particulate Matter, Fibrous Dusts and Ozone as Major Causes of Lung Carcinogenesis through Reactive Oxygen Species Mechanisms. Int. J. Environ. Res. Public Health.

[21] Laskin D.L., Sunil V.R., Gardner C.R., Laskin J.D. (2011). Macrophages and tissue injury: Agents of defense or destruction?. Ann. Rev. Pharmacol. Toxicol..

[22] Platko K., Lebeau P., Austin R.C. (2018). MAPping the kinase landscape of macrophage activation. J. Biol. Chem..

[23] Sunil V.R., Francis M., Vayas K.N., Cervelli J.A., Choi H., Laskin J.D., Laskin D.L. (2015). Regulation of ozone-induced lung inflammation and injury by the beta-galactoside-binding lectin galectin-3. Toxicol. Appl. Pharmacol..

[24] Choi J.K., Lee S.G., Lee J.Y., Nam H.Y., Lee W.K., Lee K.H., Kim H.J., Lim Y. (2005). Silica induces human cyclooxygenase-2 gene expression through the NF-kappaB signaling pathway. J. Environ. Pathol. Toxicol. Oncol..

[25] Christophi G.P., Caza T., Curtiss C., Gumber D., Massa P.T., Landas S.K. (2014). Gene expression profiles in granuloma tissue reveal novel diagnostic markers in sarcoidosis. Exp. Mol. Pathol..

[26] Kawasaki H. (2015). A mechanistic review of silica-induced inhalation toxicity. Inhal. Toxicol..

[27] Arya S.B., Kumar G., Kaur H., Kaur A., Tuli A. (2018). ARL11 regulates lipopolysaccharide-stimulated macrophage activation by promoting mitogen-activated protein kinase (MAPK) signaling. J. Biol. Chem..

[28] Guglani L., Gopal R., Rangel-Moreno J., Junecko B.F., Lin Y., Berger T., Mak T.W., Alcorn J.F., Randall T.D., Reinhart T.A. (2012). Lipocalin 2 regulates inflammation during pulmonary mycobacterial infections. PLoS ONE.

[29] Francis M., Groves A.M., Sun R., Cervelli J.A., Choi H., Laskin J.D., Laskin D.L. (2017). Editor’s Highlight: CCR2 Regulates Inflammatory Cell Accumulation in the Lung and Tissue Injury following Ozone Exposure. Toxicol. Sci..

[30] Menoret A., Kumar S., Vella A.T. (2012). Cytochrome b5 and cytokeratin 17 are biomarkers in bronchoalveolar fluid signifying onset of acute lung injury. PLoS ONE.

[31] Laskin D.L., Malaviya R., Laskin J.D. (2018). Role of Macrophages in Acute Lung Injury and Chronic Fibrosis Induced by Pulmonary Toxicants. Toxicol. Sci..

[32] Guo C., Atochina-Vasserman E., Abramova H., George B., Manoj V., Scott P., Gow A. (2016). Role of NOS2 in pulmonary injury and repair in response to bleomycin. Free Radic. Biol. Med..

[33] Zhou Y., He C.H., Yang D.S., Nguyen T., Cao Y., Kamle S., Lee C.M., Gochuico B.R., Gahl W.A., Shea B.S. (2018). Galectin-3 Interacts with the CHI3L1 Axis and Contributes to Hermansky-Pudlak Syndrome Lung Disease. J. Immunol..

[34] Bonham C.A., Strek M.E., Patterson K.C. (2016). From granuloma to fibrosis: Sarcoidosis associated pulmonary fibrosis. Curr. Opin. Pulm. Med..

[35] Patterson K.C., Strek M.E. (2013). Pulmonary fibrosis in sarcoidosis. Clinical features and outcomes. Ann. Am. Thorac. Soc..

[36] Zissel G., Prasse A., Muller-Quernheim J. (2010). Immunologic response of sarcoidosis. Semin. Respir. Crit. Care Med..

[37] Hillerdal G., Nou E., Osterman K., Schmekel B. (1984). Sarcoidosis: Epidemiology and prognosis. A 15-year European study. Am. Rev. Respir. Dis..

[38] Ungprasert P., Crowson C.S., Matteson E.L. (2016). Smoking, obesity and risk of sarcoidosis: A population-based nested case-control study. Respir. Med..

[39] Blanchet M.R., Israel-Assayag E., Cormier Y. (2004). Inhibitory effect of nicotine on experimental hypersensitivity pneumonitis in vivo and in vitro. Am. J. Respir. Crit. Care Med..

[40] Di Lorenzo G., Di Bona D., Belluzzo F., Macchia L. (2017). Immunological and non-immunological mechanisms of allergic diseases in the elderly: Biological and clinical characteristics. Immun. Ageing.

[41] Martin-Romero C., Santos-Alvarez J., Goberna R., Sanchez-Margalet V. (2000). Human leptin enhances activation and proliferation of human circulating T lymphocytes. Cell. Immunol..

[42] Mattoli S., Kleimberg J., Stacey M.A., Bellini A., Sun G., Marini M. (1997). The role of CD8+ Th2 lymphocytes in the development of smoking-related lung damage. Biochem. Biophys. Res. Commun..

